# A performance comparison of supervised machine learning models for Covid-19 tweets sentiment analysis

**DOI:** 10.1371/journal.pone.0245909

**Published:** 2021-02-25

**Authors:** Furqan Rustam, Madiha Khalid, Waqar Aslam, Vaibhav Rupapara, Arif Mehmood, Gyu Sang Choi

**Affiliations:** 1 Department of Computer Science, Khwaja Fareed University of Engineering and Information Technology, Rahim Yar Khan, Pakistan; 2 Department of Computer Science & Information Technology, The Islamia University of Bahawalpur, Bahawalpur, Punjab, Pakistan; 3 School of Computing and Information Sciences Florida International University, Miami, FL, United States of America; 4 Department of Information & Communication Engineering, Yeungnam University, Gyeongsan, Gyeongbuk, Korea; National University of Sciences and Technology, PAKISTAN

## Abstract

The spread of Covid-19 has resulted in worldwide health concerns. Social media is increasingly used to share news and opinions about it. A realistic assessment of the situation is necessary to utilize resources optimally and appropriately. In this research, we perform Covid-19 tweets sentiment analysis using a supervised machine learning approach. Identification of Covid-19 sentiments from tweets would allow informed decisions for better handling the current pandemic situation. The used dataset is extracted from Twitter using IDs as provided by the IEEE data port. Tweets are extracted by an in-house built crawler that uses the Tweepy library. The dataset is cleaned using the preprocessing techniques and sentiments are extracted using the TextBlob library. The contribution of this work is the performance evaluation of various machine learning classifiers using our proposed feature set. This set is formed by concatenating the bag-of-words and the term frequency-inverse document frequency. Tweets are classified as positive, neutral, or negative. Performance of classifiers is evaluated on the accuracy, precision, recall, and *F*_1_ score. For completeness, further investigation is made on the dataset using the Long Short-Term Memory (LSTM) architecture of the deep learning model. The results show that Extra Trees Classifiers outperform all other models by achieving a 0.93 accuracy score using our proposed concatenated features set. The LSTM achieves low accuracy as compared to machine learning classifiers. To demonstrate the effectiveness of our proposed feature set, the results are compared with the Vader sentiment analysis technique based on the GloVe feature extraction approach.

## 1 Introduction

Outbreak of Covid-19 has a socio-economic impact [1]. The World Health Organization declared it an epidemic on 30 January 2020 [2]. Since then, it has spread exponentially, inflicting serious health issues including painful deaths. As of May 31, 2020, the death toll has reached 636,633 [3]. The virus will be kept spreading in the upcoming days [4]. During the lock-down, traffic on social networking has increased tremendously [5]. Twitter has outclassed other competitors in the timely spreading of Covid-19 news [6]. Overwhelming part of these news is subjective due to the involvement of personal opinions and biasness, hence giving rise to (un)intentional fake information, uncertainty, and negativity in human social circles [7]. At the same time, this situation is catching the attention of researchers to perform computable analysis for creating a wholesome picture. This research focuses on sentiment analysis on Twitter dataset regarding Covid-19 using supervised machine learning algorithms. To this end, we undertake the following research questions:

RQ 1: How is the performance comparison of machine learning models for Covid-19 sentiment analysis on tweets?
RQ 2: Can we improve the performance of machine learning models by feature engineering?

To address these questions, the dataset is obtained from the IEEE data port. It contains the tweets IDs and sentiment scores. Corresponding to these tweets IDs, we extract tweets using an in-house built tweets crawler. The tweets are cleaned using preprocessing techniques, which include removal of non-supported information and extraction of meaningful text. Next sentiment scores are found using the TextBlob toolkit. These scores are classified as positive, negative and neutral. The dataset is split into a training set and a testing set with a ratio of 80:20 respectively. For feature extraction, techniques such as term frequency-inverse document frequency (TF-IDF), bag-of-words (BoW) and GloVe are used. We propose a feature technique by concatenating TF-IDF and BoW features. Finally, five machine learning models, random forest (RF), XGBoost classifier, support vector classifier (SVC), extra trees classifier (ETC), and decision tree (DT) are trained to test their performance on the test data. For the sake of completeness, one deep learning model, long-short term memory (LSTM) is also trained and tested. Performance is evaluated on the accuracy, precision, recall, and *F*_1_ score. We also compare our sentiment scores with those provided by the IEEE data port. The contributions of this study are:

- Using the standard feature techniques TF-IDF and BoW, performance analysis of five supervised machine learning models, RF, XGBoost, SVC, ETC, and DT for Covid-19 sentiment analysis of tweets. Additionally, we also determine the performance of the LSTM. The results are also compared with sentiment scores as provided by the IEEE data port.
- Propose a feature extraction technique based on BoW and TF-IDF and evaluate its performance.

The rest of the paper organized as follows. Section 2 contains the related work to this study. Section 3 describes the dataset and Section 4 contain methods, techniques and proposed methodology. Section 5 presents results and discussion. Section 6 gives the conclusion.

## 2 Related work

The affliction due to Covid-19 is large [8]. News about this event has dominantly surpassed other news on social media. These news also include those fake, unverified and subject to people’s biasness. Thus the call to methodically determine negativity in Covid-19 news is timely and justified.

Tweeter stays on top for gathering health-related news [9]. Sentiment analysis on Covid-19 news is done for India using a set of 24000 tweets [10]. Another study focuses on the psychological effect of Covid-19 on human behavior [11]. It reports that people are tense and their depression level increased due to Covid-19 news. Another study reports about industry crisis and new emerging opportunities due to Covid-19 [12]. There is sentiment analysis for data collected from various social networking platforms. It includes Twitter, Instagram, Reddit, YouTube, and Gab. The results report flaws in the information collected [13]. Different classifiers are tested in short text and long text information. For short text, Naive Bayes and Logistics Regression give average results of 91% and 74%. Both models do not perform well on the long text [14].

Another study about Covid-19 is done by collecting 4 million tweets from March 1, 2020, to April 21, 2020, using 25 different hashtags [15]. Thirteen topics are identified, out of which five classes are made. Latent Dirichlet Allocation (LDA) is used to recognize uni-gram, bi-gram, silent topics, themes, and sentiments in these tweets. The results are promising in the context of health-related emergency situations. Another approach detects emotions using 2500 short text ad 2500 long text messages [16]. Dominant emotion found is depression, which is understandably due to prolonged stay at home, joblessness, and fear due to virus [17]. There is also a BERT model to study emotions, which are assigned single labels and multi labels [18]. The key point of this model is to consider emojis, which is an effective way to express feelings. Another work studies Frequent pattern based sentiment analysis using the FP-growth algorithm [19]. The reported results are better than most of the other relevant works.

Lots of work is also done in the sentiment analysis domain using the deep learning approach in history [20, 21], as a study [22] proposed an approach for sentiments classification using a deep learning model. It uses NLP for topic modeling to find the core issues related to Covid-19 as expressed on social media. The classification is done using LSTM Recurrent Neural Networks (LSTM RNN) model. Another variant of the deep learning model performs sentiment analysis on Covid-19 tweets using the sentiemnt140 dataset [23]. The results show an improvement over the existing values. Another study mines a database for over 1 million tweets of five months in 2020 to assess public attitude regarding the preventative measure of mask usage during the COVID-19 pandemic [24]. It is also based on NLP and determines the frequency increase of mask-related positive tweets. Sentiment analysis is also carried out on a set of 26000 tweets in two time intervals: 1st Jan 2019 to 23rd March 2020 and December 2019 to May 2020 [25]. The set includes re-tweets. Tweets within the first interval have mostly neutral and negative polarity, while those within the second interval has neutral and positive polarity. The used classifier is based on deep learning that achieved 81% accuracy.

A model, namely BERT, uses TF-IDF for sentiment analysis and topic modeling for negative posts [26]. Statistical analysis to detect the presence of pandemic is carried out on tweets of January 2020 [27]. It used word-frequency to understand the trend and psychology of tweet users, and sentiment analysis to understand their general attitude.

In relation to all the presented works, our study focus on performance comparison of Covid-19 sentiment analysis using various machine learning algorithms. We introduce a feature set with the aim to increase accuracy. The summary of related works present in Table 1.

**Table 1:** A summary of the related work.

Ref.	Approach/Model	Aim	Dataset
[14]	Machine learning (Naïve Bayes)	Covid-19 tweets sentiment classification	Over 900000 Covid-19 tweets from February to March 2020
[22]	Deep learning (LSTM RNN)	Covid-19 reddit post sentiment classification & topic finding	Total 563,079 Covid-19 related comment from reddit
[25]	Deep learning classifier (fuzzy rule based model)	Covid-19 tweets sentiment classification	Two dataset: DATA_SET 1 226,668 tweets & DATA_SET 2 most re-tweeted tweets (23000)
[28]	Deep learning (ERNIE & BiLSTM + attention + CRF)	Covid-19 blog post sentiment classification for Chinese Text	60000 micro-blog Chinese text post related COVID-19
[29]	Lexicon-based technique ( Word-Emotion Lexicon)	Covid-19 news headline sentiments and emotions classification	Total 141,208 news headlines of global English news sources
[23]	Deep learning (Deep LSTM)	Covid-19 tweets cross-cultural polarity and emotion detection	Total 460,286 Covid-19 related tweets
[24]	Natural Language Processing Techniques	Topical sentiment analysis for Covid-19 tweets	Over 1 million Covid-19 mask-related tweets
[26]	Machine learning (BERT model with TF-IDF features)	Topic discovering behind Covid-19 negative tweets	Total 999978 randomly selected COVID-19 related Weibo posts

## 3 Dataset description

The dataset we used in this study is obtained from the IEEE data port on May 31, 2020 [30]. It contains the tweet IDs and sentiment scores of 7528 tweets. A sample data is shown in Table 2. Tweets IDs are extracted using the following filters: language “en” and keywords “corona”, “coronavirus”, “covid”, “pandemic” and variants such as “sarscov2”, “nCov”, “covid-19”, “ncov2019”, “2019ncov” and their hashtags. IEEE data port does not provide tweet text, resorting us to develop an in-house crawler that could extract them from Tweeter based on the tweet IDs. Since IEEE data port provides tweet IDs the next day, our dataset corresponds to the tweets of May 30, 2020. After preprocessing the data, we find sentiment scores using the TextBlob. The results are shown in Table 3.

**Table 2:** A sample of the dataset available at IEEE data port [30].

Tweet ID	Sentiment Score
1266588391854030000	-0.0571428571428571
1266588391858200000	-0.4
1266588392109760000	0.25

**Table 3:** A comparison of sentiment scores between those provided by IEEE data port (SS1) and those determined by our approach after preprocessing (SS2).

ID	Tweets	SS1	SS2
1266588393594650000	RT @ziwe: caná€™t believe corona blew a 28-3 lead to racism	0	0
1266588391858200000	@whozak @bonifacemwangi She can’t walk but can dance oops @AtwoliDza is cursed but anyway biwott was also there andá€¦ https://t.co/7knyPDDh06	-0.4	0
1266588392109760000	RT @FLOTUS: Our country allows for peaceful protests, but there is no reason for violence. Iá€™ve seen our citizens unify & take care of oneá€¦	0.25	0.25

In Table 3, SS1 and SS2 represent sentiment scores that is provided by IEEE data port and that is found by us after preprocessing the data. The sentiment scores are classified as positive (greater than 0), neutral (equal to 0) and negative (less than 0). Table 4 shows the counts of tweets present in these classes.

**Table 4:** Counts of tweets within the three classes.

Sentiment	SS1	SS2
Neutral	2678	2938
Positive	2227	2293
Negative	2623	2297
Total	7528	7528

**Fig 1:**
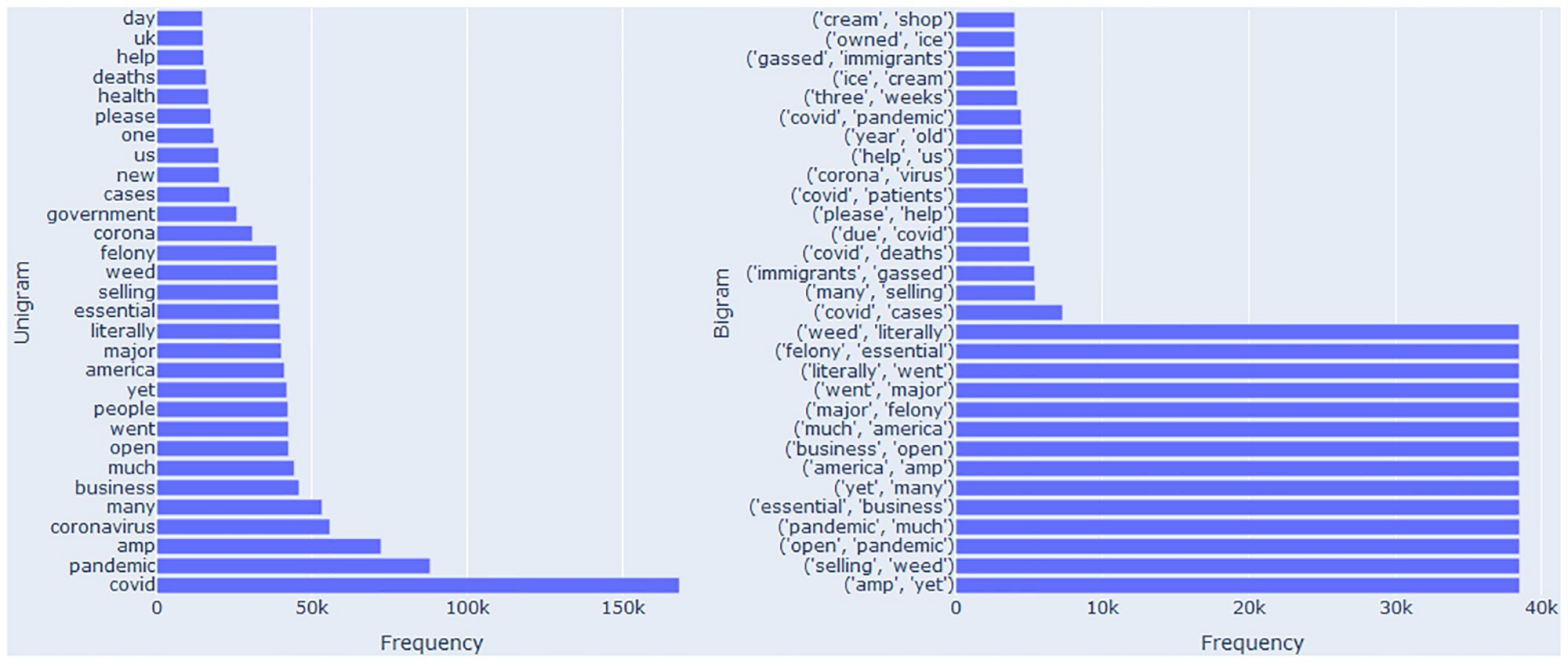
Term counts in Covid-19 tweets dataset [30].

**Fig 2:**
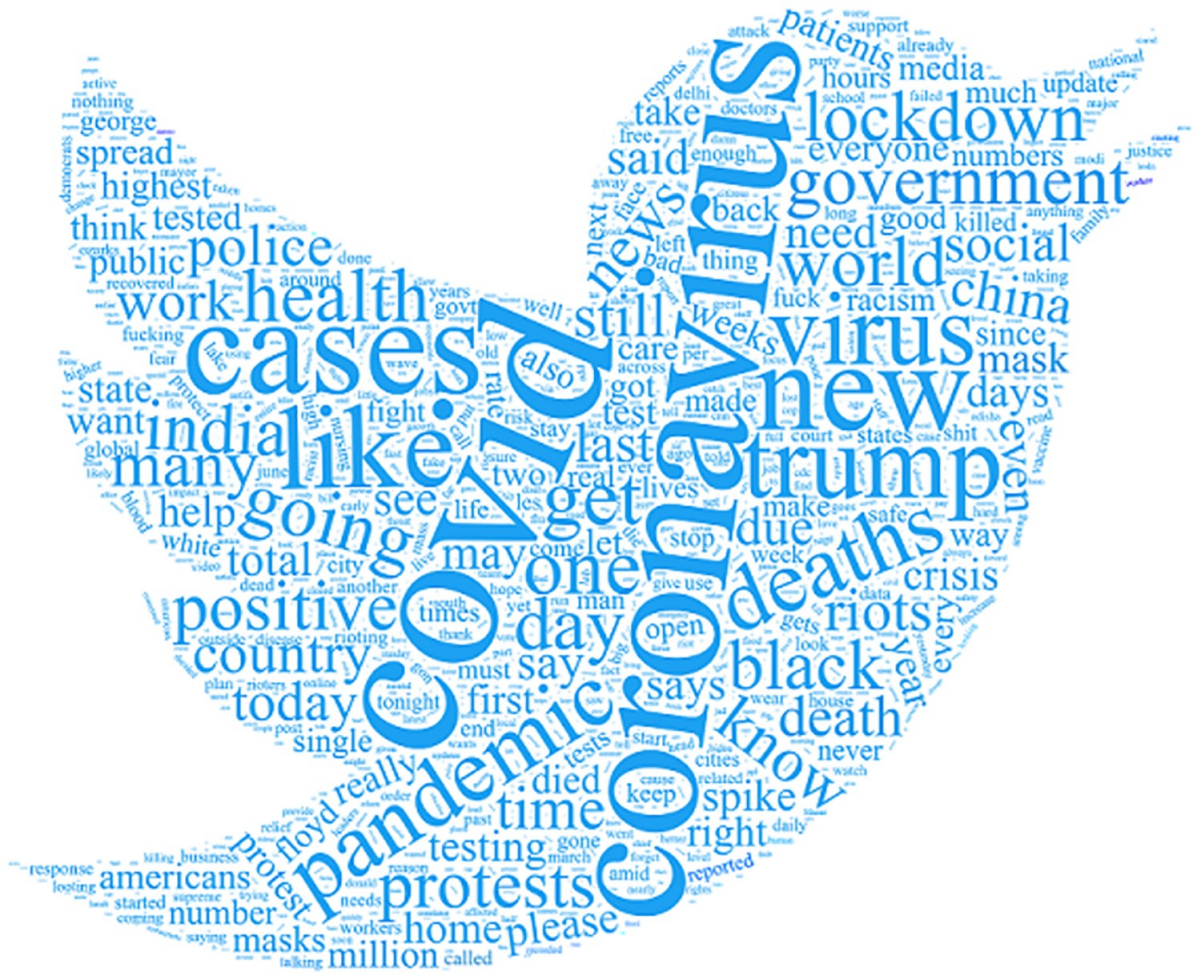
The word-cloud to highlight the topics after preprocessing.

Fig 1 shows the counts of most frequent uni-gram and bi-gram terms as provided by IEEE data port and Fig 2 shows the most frequent words as taken from tweets. Figs 1 and 2 also depict the topics used in tweets. They are ‘conronavirus’, ‘covid’ ‘trump’ ‘deaths’ ‘lockdown’, ‘government’ and ‘cases’. These words indicate that people are talking about government polices for Covid-19, and lockdown/deaths because of Covid-19.

## 4 Methods and proposed approach

The Lexicon based machine learning models are defined next.

### 4.1 Supervised machine learning method

We use five supervised machine learning models, their parameters settings are given in in Table 5.

**Table 5:** The parameter settings of five machine learning models.

Classifier	Parameters
RF	n_estimator = 300, max_depth = 300, random_state = 27
XGBoost	n_estimator = 300, learning_rate = 0.2, max_depth = 300, random_state = 27
SVC	Kernel = sigmoid, C = 3.0, random_state = 27
ETC	n_estimator = 300, max_depth = 300, random_state = 27
DT	max_depth = 300, criterion = gini

#### 4.1.1 RF

RF is used for both classification and regression problems [31]. RF is an ensemble model that uses bagging techniques. It generates several trees and performs voting between them to make a majority decision. Prediction accuracy increases with the number of tree. RF reduces the problem of over-fitting by using a bootstrap sampling technique [32]. RF can be defined as the mode of the multiple prediction trees, mode(t1, t2, t3,…, tn). Here t1, t2, t3 and tn are the predicted values against a tweet, whose Mode is taken. RF parameter settings are given in Table 5. By setting n_estimator = 300, we generate 300 decision trees. By setting max_depth = 300, the tree is restricted to grow to a maximum depth of 300 levels, thus effectively reducing the complexity of the decision tree.

#### 4.1.2 XGBoost

XGBoost (eXtreme Gradient Boosting) works the same way as Gradient Boosting classifier but with an extra feature of assigning weight to each sample as in Adaboost classifier [33, 34]. XGBoost is a tree-based model which gained lots of popularity in recent times. It fits a number of weak learners (decision trees) parallelly unlike gradient boosting which does this sequentially. Due to this, XGBoost gives a speed boost. XGBoost has regularization techniques to control over-fittings such as L1 and L2 but these techniques are not available in Gradient Boosting and Adaboost classifiers. Another key feature of XGBoost is scalability, which means that it also can perform better on a distributed system and can process larger datasets. It uses a Log Loss function, which is also helpful to minimize the loss and improve accuracy. The Log Loss function considers the probability of false classifications [35]. This loss function can be defined as

$$\begin{array}{c} \log loss=\;-\frac{1}{N}\sum_{i=1}^{N}y_{i}.\log\left(p\left(y_{i}\right)\right)+\left(1-y_{i}\right).\log\left(1-p\left(y_{i}\right)\right). \end{array}$$ (1)

We set four parameters of XGBoost as given in Table 5. The n_estimator = 300 implies XGBoost uses 300 decision trees as a base learner, which will take part in the prediction process. The parameter max_depth = 300 restricts the growth of trees to a maximum 300. The learning_rate = 0.2 helps to control the over-fitting in the model [34]. The random_state = 27 controls the random seed given to each Tree estimator at each boosting iteration. In addition, it controls the random permutation of the features at each split.

#### 4.1.3 SVC

SVC is a linear model and used for sentiment analysis in many research work [36–38]. SVC maps each item as a data point in an n-dimensional space, where n is the number of features. It performs classification by finding the “best fit” hyper-plane that can differentiate between the classes. We use SVC with a sigmoid kernel and use another parameter C = 3.0 as a regularization value, as given in Table 5.

#### 4.1.4 ETC

ETC implements the meta-estimator, which trains/fits the number of weak learners (randomized decision trees) on various samples of the dataset and boosts the prediction accuracy [39, 40]. It is also an ensemble learning model used for classification purposes like RF, thus it is considered similar to RF. The only difference between ETC and RF is the way trees are constructed in the forest. ETC generates decision trees on the original training sample, while RF constructs decision trees on the bootstrap samples drawn from the original dataset. At each test node, each tree is provided with a random sample of k features drawn from the feature-set. Each decision tree must select the best feature to split the data based on some mathematical criteria (typically Gini Index). This random sample of features leads to the creation of multiple de-correlated decision trees. We use ETC with two main parameters, n_estimators = 300 and max_depth = 300, as given in Table 5.

#### 4.1.5 DT

DT acquires information in the form of a tree, which can also be rewritten as a set of discrete rules [41, 42]. The key advantage of DT is the use of decision rules and feature subsets that appear at various classification stages. A DT consists of different types of nodes such as a leaf node and a number of internal nodes with branches. Each leaf node represents a class corresponding to an example while each internal node represents features and branches representing the conjunction of features that lead towards classification. The performance of DT depends on how well it is constructed on the training set. We use the parameter max_depth = 300 to restrict DT to grow a maximum depth of 300 as given in Table 5.

### 4.2 TextBlob

TextBlob is a Python library that is used in natural language processing (NLP) tasks such as part-of-speech tagging, sentiment analysis, noun phrase extraction, translation, classification and many more [43, 44]. We use TextBlob to find the sentiments from Covid-19 tweets. TextBlob sentiment function returns two properties against each tweet, polarity score in the range [-1,1] and subjectivity score in the range [0, 1]. Negative, zero and positive polarity scores represent negative, neutral and positive statements, respectively. Subjectivity refers to the expression of opinions, evaluations, feelings and speculations [45].

### 4.3 Feature extraction techniques

In order to use the machine learing models, we need to extract features of tweets. Two feature extraction techniques, BoW and TF-IDF are used.

#### 4.3.1 TF-IDF

TF-IDF is a feature extraction technique used for text analysis tasks [28–38]. It gives weighted features for performance boosting [31, 39, 46]. TF-IDF finds the weight of each feature in a document using the product of term frequency (TF) and inverse document frequency (IDF). TF is the frequency of a feature in document and depends on the length of the document. It can be defined as

$$\begin{array}{c} tf_{t,d}=\frac{count_{t,d}}{totalcount_{d}}, \end{array}$$ (2)

where *count*_(_ *t*, *d*) is the number of term *t* in the document d and *totalcount*_*d*_ is the total number of all terms in the document *d*. IDF measures the extent of a term t being informative in a document for model training. It can be computed as

$$\begin{array}{c} idf\;=\;N/Df_{t}, \end{array}$$ (3)

where *N* is the number of documents in the corpus and *Df*_*t*_ is the number of documents that contain the term *t*. IDF measures the weight of a term t low when term t occurs frequently in many documents. For instance, stopwords have low IDF value. Finally, TF-IDF can be defined as

$$\begin{array}{c} tf-idf\;=\;tf_{t,d}\;*\;\log\left(idf\right). \end{array}$$ (4)

#### 4.3.2 BoW

The BoW is the simplest feature extraction technique used in information retrieval and NLP tasks [31, 47]. BoW is mostly used in text classification tasks where the occurrence of each word in a document is used for model training. BoW generates the vocabulary all the uniques words and their occurrence frequencies in the all documents for the training of learning models.

#### 4.3.3 Concatenation of BoW and TF-IDF

**Fig 3:**
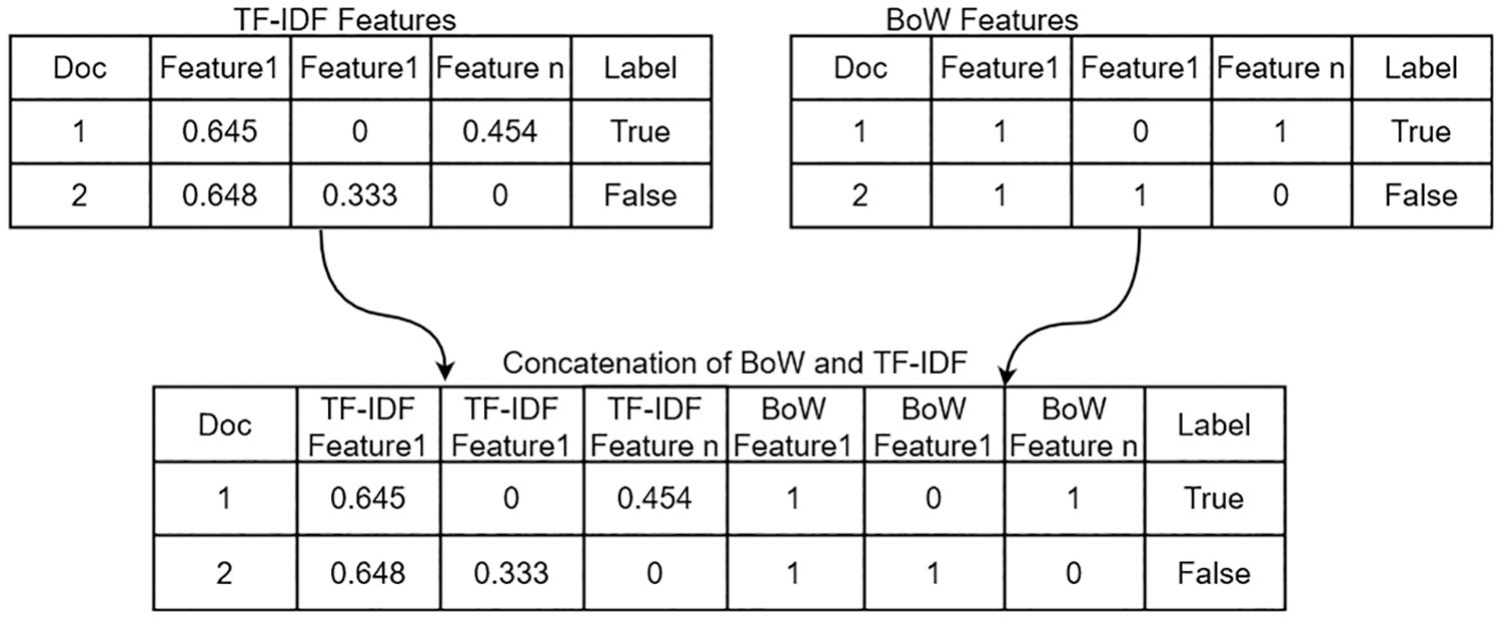
Our proposed approach based on concatenation of BoW and TF-IDF.

To boost the performance of machine learning models, we propose a concatenation of BoW and TF-IDF features, as shown in Fig 3. Such concatenation is helpful for learning models to boost their accuracies.

### 4.4 Evaluation parameters

To evaluate the performance of the machine learning models, we have used four evaluation parameters, accuracy score, precision score, recall score and *F*_1_ score. The accuracy score is the fraction of correct predictions. The maximum accuracy score can be 1 and the minimum accuracy score can be 0. It is given as

$$\begin{array}{c} accuracy=\;\frac{TP+TN}{TP+TN+FP+FN}. \end{array}$$ (5)

Four basic notations are explained as follows.

**True Positives (TP):** The number of correct positive predictions of a class.
**True Negatives (TN):** The number of correct negative predictions of a class.
**False Positives (FP):** The number of incorrect positive predictions of a class.
**False Negatives (FN):** The number of incorrect negative predictions of a class.

Precision indicates the exactness of the classifiers. It lies in [0, 1] and calculated as

$$\begin{array}{c} precision=\frac{TP}{\left(TP+FP\right)}. \end{array}$$ (6)

Recall indicates about the completeness of a classifier. It lies in [0, 1] and calculated as

$$\begin{array}{c} recall=\frac{TP}{\left(TP+FN\right)}. \end{array}$$ (7)

*F*_1_ score is a harmonic mean of precision and recall scores. It lies in [0, 1] and calculated as

$$\begin{array}{c} F_{1}=2*\frac{\left(precision\;*\;recall\right)}{\left(precision\;+\;recall\right)}. \end{array}$$ (8)

### 4.5 Proposed methodology

**Fig 4:**
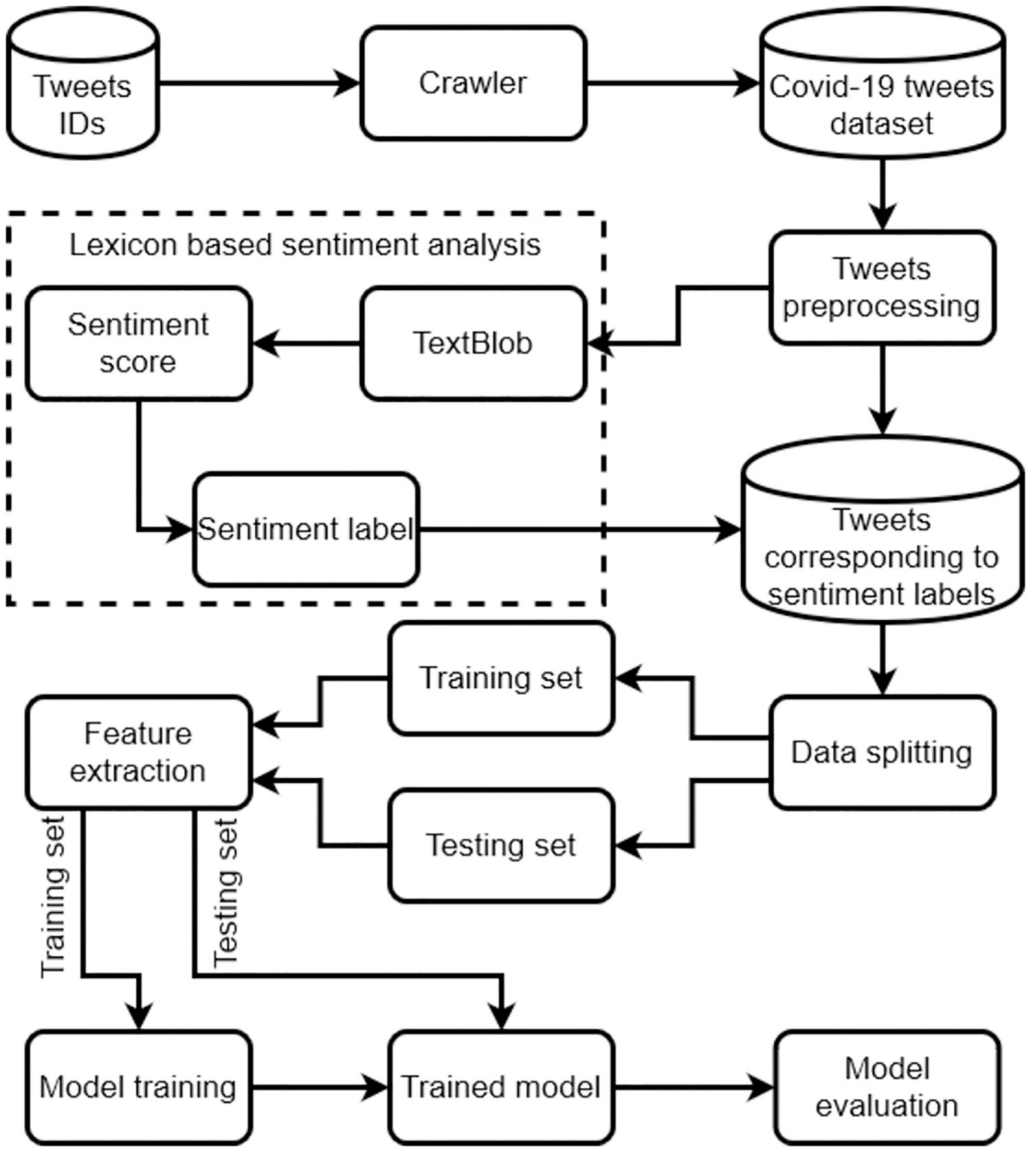
Our proposed methodology.

A thematic of the our proposed methodology is shown in Fig 4. After extracting tweets using our in-house built crawler, data is preprocessed. Preprocessing is an important step, which affects accuracy of learning models. We remove stopwords, usernames, link punctuation’s and numeric values from tweets and apply stemming techniques. A sample data is given in Table 6. After preprocessing, the results are given in Tables 7–10.

**Table 6:** Three sample tweets.

No.	Tweets
1.	RT @skaijackson: Donald Trump called the coronavirus the Chinese virus
2.	RT @latimes: owners go to jail for breaking coronavirus rules? https://t.co/tltJJvIT8R
3.	RT bryanbehar: 40 million out of work #covid

**Table 7:** Sample tweets after removing usernames and links.

Tweets Before Removal	Tweets After Removal of Usernames and Links
RT @skaijackson: Donald Trump called the coronavirus the Chinese virus	Donald Trump called the coronavirus the Chinese virus
RT @latimes: owners go to jail for breaking coronavirus rules? https://t.co/tltJJvIT8R	owners go to jail for breaking coronavirus rules?
RT bryanbehar: 40 million out of work #covid	40 million out of work #covid

**Table 8:** Sample tweets after removing punctuation marks and conversion to lower case.

Tweets Before Removal	Tweets After Removal of Punctuation Marks and Conversion to Lower Case
Donald Trump called the coronavirus the Chinese virus	donald trump called the coronavirus the chinese virus
owners go to jail for breaking coronavirus rules?	owners go to jail for breaking coronavirus rules
40 million out of work covid	40 million out of work covid

**Table 9:** Sample tweets after removing stopwords and numeric values.

Tweets Before Removal	Tweets After Removal of Stopwords and Numeric values
donald trump called the coronavirus the chinese virus	donald trump called coronavirus chinese virus
owners go to jail for breaking coronavirus rules	owners go jail breaking coronavirus rules
40 million out of work covid19	million out work covid19

**Table 10:** Sample tweets after stemming.

Tweets Before Stemming	After Stemming
donald trump called coronavirus chinese virus	donald trump call coronaviru chines viru
owners go jail breaking coronavirus rules	owner go jail break coronaviru rule
million out work covid19	million out work covid19

#### 4.5.1 Removal of usernames and links

Usernames (e.g. @username) and links in tweets do not contribute to sentiment analysis, hence they need to be removed. We also remove special stopwords ‘RT’ from tweets, which are not important for topic analysis [48]. The results are given in Table 7.

#### 4.5.2 Removal of punctuation marks and conversion to lower case

Punctuation characters hinder machine understanding, hence they are removed, so do hashtag markers ‘#’. Similarly all terms are converted to lower case for uniformity. For instance, “COVID”, “Covid”, “covid”, and #covid are all converted to “covid” because machine learning models are case sensitive. The results are given in Table 8.

#### 4.5.3 Removal of stopwords and numeric values

We remove commonly used stopwords such as a, an, as and etc. to reduce the noise. We also remove the numeric values such as 123, which are not valuable for text analysis. Alpha-numeric words such as covid19 are not removed because most of them are technical terms. The results are given in Table 9.

#### 4.5.4 Stemming

Stemming is also an important technique in text preprocessing to reduce the terms to their root form [49]. For instance, machine learning algorithms consider “go”, “goes” and “going” as different features even if they contain the same information. Reaching the stem reduces the complexity in the text features [50]. The results are given in Table 10.

After preprocessing, we apply the TextBlob technique on the dataset to find sentiment scores. Due to cleaning noise, the TextBlob performs better than the original dataset. A performance comparison of the TextBlob sentiment scores between the original data and the preprocessed data is given in Tables 11 and 12. Please note the change in labels.

**Table 11:** The TextBlob performance on original data.

Tweet ID	Tweet Text	Score	Label
1266588391858208770	@whozak @bonifacemwangi She can’t walk but can dance oops @AtwoliDza is cursed but anyway biwott was also there and … https://t.co/7knyPDDh0	-0.4	Negative
1266588393162575872	Ppl not scared of COVID-19 no more? Our numbers definitely going back up next week.	-0.0625	Negative

**Table 12:** TextBlob performance after preprocessed data.

Tweet ID	Tweet Text	Score	Label
1266588391858208770	walk dance oops cursed anyway biwott also	0.0000	Neutral
1266588393162575872	ppl scare covid number definitely go back next week	0.0000	Neutral

After finding sentiment scores, the dataset is split into a training set and a testing set with a ratio of 80:20 as shown in Table 13. Features TF-IDF, BoW, and concatenation of TF-IDF and BoW are used for the training of learning models. For demonstration purposes, we apply these feature engineering techniques on two sample tweets ‘trump call covid chines viru’ and ‘covid could kill’. The results are given in Tables 14–16.

**Table 13:** Training and testing tweets count for SS1 and SS2.

Sentiment	SS1			SS2		
	Training set	Testing Set	Total	Training set	Testing Set	Total
Neutral	2145	533	2678	2340	598	2938
Positive	1783	444	2227	1844	449	2293
Negative	2094	529	2623	1838	459	2297
Total	6022	1506	7528	6022	1506	7528

**Table 14:** TF-IDF features on two sample tweets.

Doc	call	chines	could	covid	kill	trump	viru
1	0.471	0.471	0	0.335	0	0.471	0.471
2	0	0	0.631	0.449	0.631	0	0

**Table 15:** BoW features on two sample tweets.

Doc	call	chines	could	covid	kill	trump	viru
1	1	1	0	1	0	1	1
2	0	0	1	1	1	0	0

**Table 16:** Concatenated features on two sample tweets.

Doc	call	chines	could	covid	kill	trump	viru	call	chines	could	covid	kill	trump	viru
1	0.471	0.471	0	0.335	0	0.471	0.471	1	1	0	1	0	1	1
2	0	0	0.631	0.449	0.631	0	0	0	0	1	1	1	0	0

0: 2145, 1: 1783, 2: 2094)

After training the learning models, the performance is evaluated on accuracy score, precision score, recall score and *F*_1_ score (see Section 4.4).

## 5 Results

This section presents the Covid-19 sentiment analysis results of the machine learning models RF, XGBoost, SVC, ETC, and DT using features BoW, TF-IDF, and concatenation of TF-IDF and BoW. We compare the model performance between SS1 and SS2. In results tables and confusion matrices, 0, 1, 2 represent neutral, positive, and negative sentiments respectively.

### 5.1 Results with TF-IDF

The TF-IDF results of used models are given Tables 17 and 18. ETC model performs better for both SS1 and SS2 using TF-IDF. It has a higher accuracy score 0.92 for SS2. It is due to the fact that TextBlob performs better when given a cleaned data. Both SS1 and SS2, Tree-based models give better performance for both SS1 and SS2.

**Table 17:** Models performance for SS1 using TF-IDF.

Model	Accuracy	Class	Precision	Recall	*F*_1_ score
RF	0.86	0	0.76	0.96	0.85
		1	0.92	0.76	0.83
		2	0.95	0.84	0.89
		macro avg	0.88	0.85	0.86
		weighted avg	0.87	0.86	0.86
XGboost	0.85	0	0.78	0.89	0.83
		1	0.87	0.79	0.82
		2	0.91	0.85	0.88
		macro avg	0.85	0.84	0.85
		weighted avg	0.85	0.85	0.85
SVC	0.85	0	0.81	0.86	0.84
		1	0.81	0.82	0.82
		2	0.92	0.85	0.88
		macro avg	0.85	0.85	0.85
		weighted avg	0.85	0.85	0.85
ETC	0.88	0	0.80	0.98	0.88
		1	0.92	0.79	0.85
		2	0.96	0.85	0.90
		macro avg	0.89	0.87	0.88
		weighted avg	0.89	0.88	0.88
DT	0.83	0	0.78	0.89	0.83
		1	0.84	0.75	0.79
		2	0.89	0.85	0.87
		macro avg	0.84	0.83	0.83
		weighted avg	0.84	0.83	0.83

**Table 18:** Models performance for SS1 using TF-IDF.

Model	Accuracy	Class	Precision	Recall	*F*_1_ score
RF	0.90	0	0.84	0.99	0.91
		1	0.94	0.83	0.88
		2	0.97	0.86	0.91
		macro avg	0.92	0.89	0.90
		weighted avg	0.91	0.90	0.90
XGboost	0.90	0	0.84	0.97	0.90
		1	0.93	0.84	0.89
		2	0.95	0.86	0.90
		macro avg	0.91	0.89	0.90
		weighted avg	0.90	0.90	0.90
SVC	0.89	0	0.86	0.93	0.90
		1	0.89	0.89	0.89
		2	0.96	0.86	0.91
		macro avg	0.89	0.89	0.89
		weighted avg	0.89	0.89	0.89
ETC	0.92	0	0.88	0.99	0.93
		1	0.95	0.87	0.91
		3	0.97	0.89	0.93
		macro avg	0.93	0.92	0.92
		weighted avg	0.93	0.92	0.92
DT	0.89	0	0.88	0.92	0.90
		1	0.89	0.86	0.87
		2	0.90	0.88	0.89
		macro avg	0.89	0.89	0.89
		macro avg	0.89	0.89	0.89

**Fig 5:**
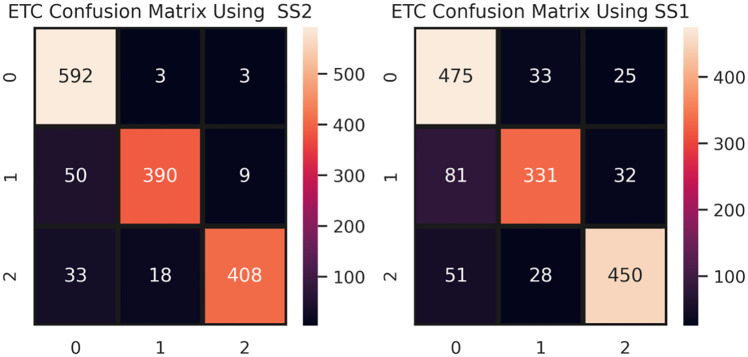
ETC confusion matrix using SS1 and SS2 under TF-IDF features.

The confusion matrix given in Fig 5 shows the performance of the ETC model. Based on our chosen ratio of 80:20 for training data and testing data, there are 7528 tweets and 1506 tweets, respectively. ETC correctly predicts 1390 tweets for SS2, while it makes 116 incorrect predictions. For SS1, ETC makes 250 incorrect predictions.

### 5.2 Results with bag-of-words

The BoW results of used models are given Tables 19 and 21. BoW results are comparable to that of TF-IDF. We observe that tree-based models RF, XGBoost, and DT perform slightly better for BoW than TF-IDF, while SVC perform better for TF-IDF. ETC performance for SS2 is the same for both BoW and TF-IDF.

**Table 19:** Models performance for SS1 using BoW.

Model	Accuracy	Class	Precision	Recall	*F*_1_ score
RF	0.87	0	0.77	0.98	0.86
		1	0.92	0.77	0.84
		2	0.96	0.84	0.90
		macro avg	0.89	0.86	0.87
		macro avg	0.88	0.87	0.87
XGboost	0.86	0	0.80	0.90	0.85
		1	0.89	0.79	0.84
		2	0.90	0.86	0.88
		macro avg	0.86	0.85	0.86
		macro avg	0.86	0.86	0.86
SVC	0.82	0	0.75	0.90	0.82
		1	0.84	0.74	0.79
		2	0.91	0.82	0.86
		macro avg	0.83	0.82	0.82
		weighted avg	0.83	0.82	0.82
ETC	0.88	0	0.79	0.98	0.87
		1	0.93	0.78	0.85
		2	0.95	0.85	0.90
		macro avg	0.89	0.87	0.87
		weighted avg	0.89	0.88	0.88
DT	0.85	0	0.81	0.89	0.85
		1	0.86	0.81	0.83
		2	0.90	0.85	0.87
		macro avg	0.86	0.85	0.85
		weighted avg	0.86	0.85	0.85

**Fig 6:**
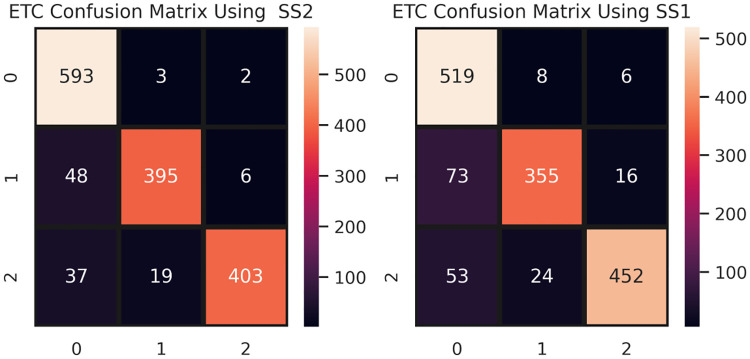
ETC confusion metric for SS1 and SS2 under BoW features.

Referring to results given in Tables 19 and 20, we observe that the tree-based models RF, XGBoost, and DT perform well. ETC achieved high accuracy levels of 0.92 and 0.88 for SS2 and SS1, respectively. Tree-based models also achieved good accuracy of 0.91. The performance ETC is also elaborated by the confusion matrices, shown in Fig 6. ETC makes more correct predictions when we train the model with the clean data. Out of 1506 predictions, it makes 1390 correct predictions for SS2, which are only 1326 for SS1.

**Table 20:** Models performance for SS2 using BoW.

Model	Accuracy	Class	Precision	Recall	*F*_1_ score
RF	0.91	0	0.86	0.99	0.92
		1	0.95	0.85	0.90
		2	0.97	0.87	0.92
		macro avg	0.93	0.90	0.91
		weighted avg	0.92	0.91	0.91
XGboost	0.91	0	0.87	0.95	0.91
		1	0.94	0.87	0.90
		2	0.94	0.89	0.92
		macro avg	0.92	0.91	0.91
		weighted avg	0.91	0.91	0.91
SVC	0.87	0	0.82	0.94	0.88
		1	0.88	0.80	0.84
		2	0.93	0.84	0.88
		macro avg	0.88	0.86	0.87
		weighted avg	0.87	0.87	0.87
ETC	0.92	0	0.88	0.99	0.93
		1	0.95	0.88	0.91
		2	0.98	0.88	0.93
		macro avg	0.93	0.92	0.92
		weighted avg	0.93	0.92	0.92
DT	0.91	0	0.92	0.97	0.94
		1	0.93	0.91	0.92
		2	0.93	0.90	0.92
		macro avg	0.93	0.93	0.93
		weighted avg	0.93	0.93	0.93

### 5.3 TF-IDF & BoW concatenation

We concatenated TF-IDF and BoW features with the aim to achieve high accuracy of machine learning models (see Section 4.3.3). The results are given in Tables 21 and 22. Overall the performance is boosted with the combination. ETC accuracy improves to 0.93 when we train it using clean data. XGBoost and RF also outperform their previous results. The performance of ETC and XGBoost is also improved for SS1.

**Table 21:** Models performance for SS1 using TF-IDF and BoW concatenation.

Model	Accuracy	Class	Precision	Recall	*F*_1_ score
RF	0.88	0	0.78	0.97	0.87
		1	0.93	0.79	0.85
		2	0.96	0.84	0.90
		macro avg	0.89	0.87	0.87
		weighted avg	0.88	0.88	0.88
XGboost	0.89	0	0.85	0.95	0.89
		1	0.92	0.83	0.88
		2	0.93	0.86	0.90
		macro avg	0.89	0.89	0.89
		weighted avg	0.89	0.89	0.89
SVC	0.86	0	0.81	0.91	0.85
		1	0.85	0.80	0.83
		2	0.93	0.85	0.89
		macro avg	0.86	0.85	0.85
		weighted avg	0.86	0.86	0.86
ETC	0.89	0	0.80	0.98	0.89
		1	0.94	0.79	0.86
		2	0.95	0.86	0.90
		macro avg	0.89	0.88	0.89
		weighted avg	0.89	0.89	0.89
DT	0.82	0	0.79	0.86	0.82
		1	0.83	0.74	0.78
		2	0.85	0.84	0.85
		macro avg	0.82	0.82	0.82
		weighted avg	0.82	0.82	0.82

**Table 22:** Models performance for SS2 using TF-IDF and BoW concatenation.

Model	Accuracy	Class	Precision	Recall	*F*_1_ score
RF	0.92	0	0.87	0.99	0.93
		1	0.95	0.85	0.89
		2	0.96	0.87	0.92
		macro avg	0.92	0.91	0.92
		weighted avg	0.92	0.92	0.92
XGboost	0.92	0	0.88	0.95	0.91
		1	0.95	0.88	0.91
		2	0.95	0.90	0.92
		macro avg	0.93	0.92	0.92
		weighted avg	0.92	0.92	0.92
SVC	0.89	0	0.85	0.94	0.88
		1	0.91	0.84	0.86
		2	0.92	0.85	0.88
		macro avg	0.89	0.88	0.89
		weighted avg	0.89	0.89	0.89
ETC	0.93	0	0.89	0.93	0.91
		1	0.90	0.86	0.88
		2	0.91	0.88	0.90
		macro avg	0.90	0.89	0.89
		weighted avg	0.90	0.90	0.90
DT	0.91	0	0.92	0.97	0.94
		1	0.93	0.91	0.92
		2	0.93	0.90	0.92
		macro avg	0.93	0.93	0.93
		weighted avg	0.93	0.93	0.93

**Fig 7:**
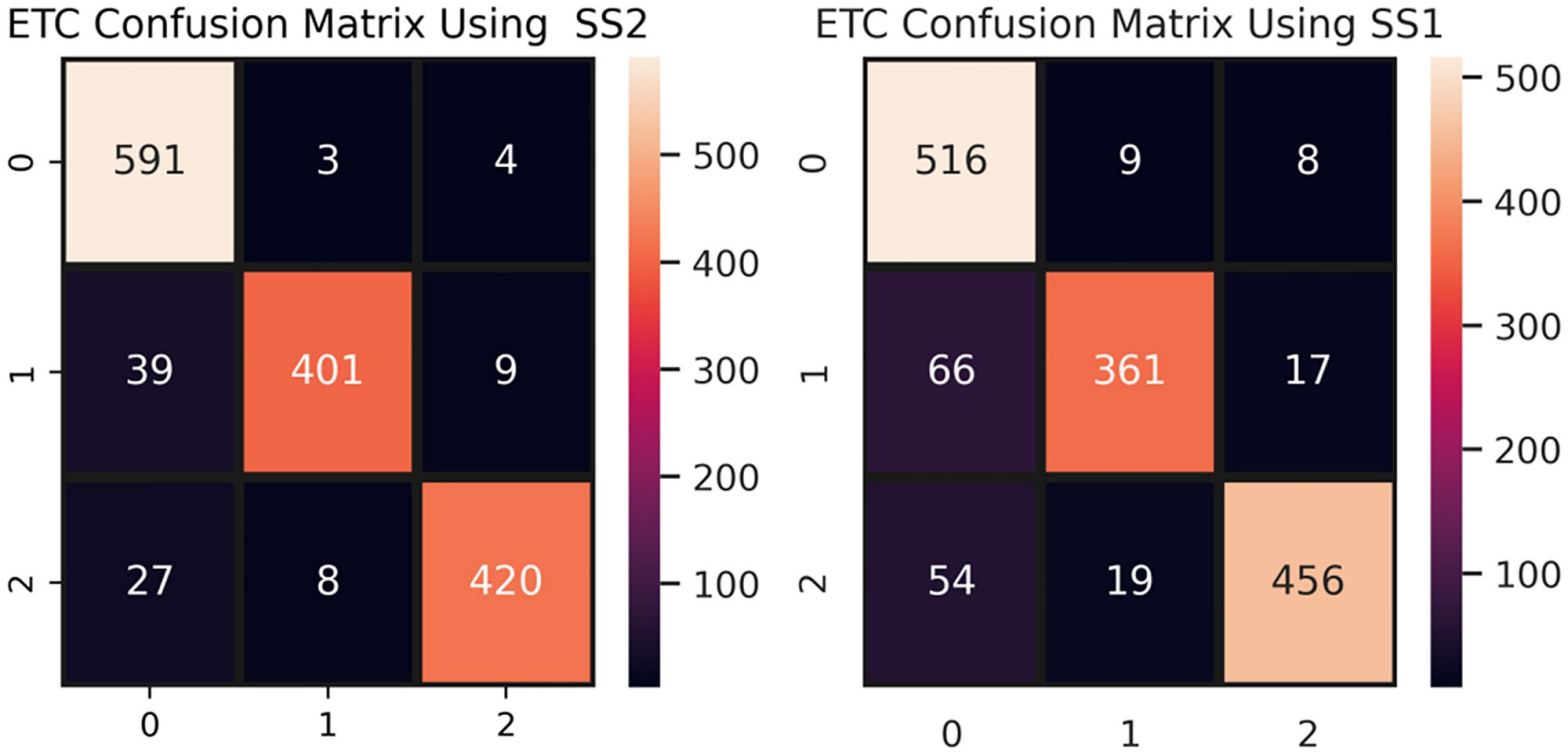
ETC confusion matrix for SS1 and SS2 using concatenated features.

The confusion matrix of the best performer, ETC is shown in Fig 7 for the concatenated features. ETC makes 1412 correct predictions out of 1506, and only 94 incorrect, which is the lowest ratio of incorrect predictions in this study. The improvement in results is due to the concatenated features. By concatenation, the feature set size increases so the model has more features to learn and to improve its accuracy. As is observed in the SS2 case, ETC gives 94 incorrect predictions. Most of these incorrect predictions are made for the positive class, which is 39 out of 94. The reason behind this observation is that the positive class has fewer examples in SS2 as compared to other classes. Thus the model has a low positive class example ratio for training as compare to the other classes.

### 5.4 Comparison of our proposed approach with other techniques

In this section, we compare the results of concatenated features with Vader [51] and GloVe [52] to show the significance of this study. Vader (Valence Aware Dictionary and sEntiment Reasoner) is a lexicon and rule-based technique to find the sentiment score from the social media platforms [51]. It also works well on text from other domains. It assigns intensity to each word in the tweets and then sum-up all the intensities to obtain the sentiment score. The selection of Vader for comparison is due to the fact that many research studies have shown the better performance of Vader on social media type text [53, 54]. Results given in Table 23 show that SS2 accuracy outperforms Vader in all cases, thus the significance of SS2.

**Table 23:** Models accuracy performance comparison for SS2 and Vader.

Model	BoW		TF-IDF		Concatenated Features	
	SS2	Vader	SS2	Vader	SS2	Vader
RF	0.91	0.84	0.90	0.84	0.92	0.85
XGboost	0.91	0.85	0.90	0.83	0.92	0.85
SVC	0.87	0.80	0.89	0.86	0.89	0.81
ETC	0.92	0.86	0.92	0.86	0.93	0.87
DT	0.91	0.85	0.89	0.82	0.91	0.83

**Fig 8:**
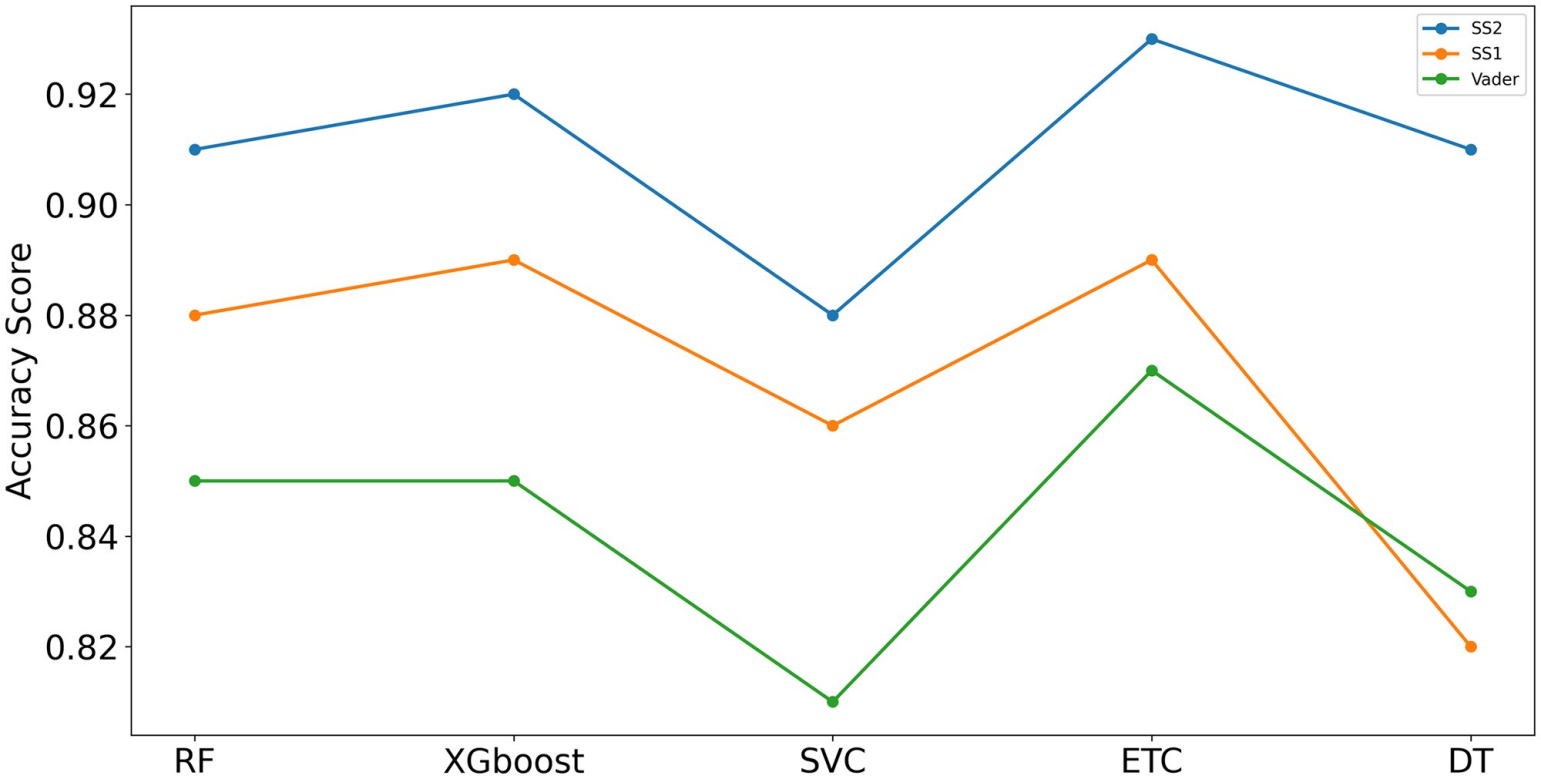
Models accuracy performance for SS1, SS2 and Vader using concatenated feature engineering technique.

Fig 8 shows the performance comparison between the models for SS1, SS2 and Vader using concatenated features. SS2 accuracy is highest as compared to SS1 and Vader.

Similarly, results are compared with GloVe (Global Vector), which is an unsupervised learning algorithm to obtain vector representations of any word. It creates a feature matrix on the basis of feature-feature co-occurrence [52]. Selecting GloVe to compare with our approach to existing approaches is due to the fact that it is mostly used for feature extraction [55, 56]. We also selected two deep learning approaches to extract the features that are used to train our machine learning models: a combination of CNN and LSTM (CNN-LSTM) and Deep Neural Networks (DNN). The resultant performance is not prominent, as given in Table 24.

**Table 24:** Model accuracy performance comparison on SS2 with GloVe, CNN-LSTM, DNN and concatenated features.

Model	GloVe	DNN	CNN-LSTM	Concatenated Features
RF	0.81	0.78	0.79	0.92
XGboost	0.80	0.77	0.76	0.92
SVC	0.70	0.66	0.52	0.89
ETC	0.80	0.78	0.79	0.93
DT	0.78	0.75	0.73	0.91

**Fig 9:**
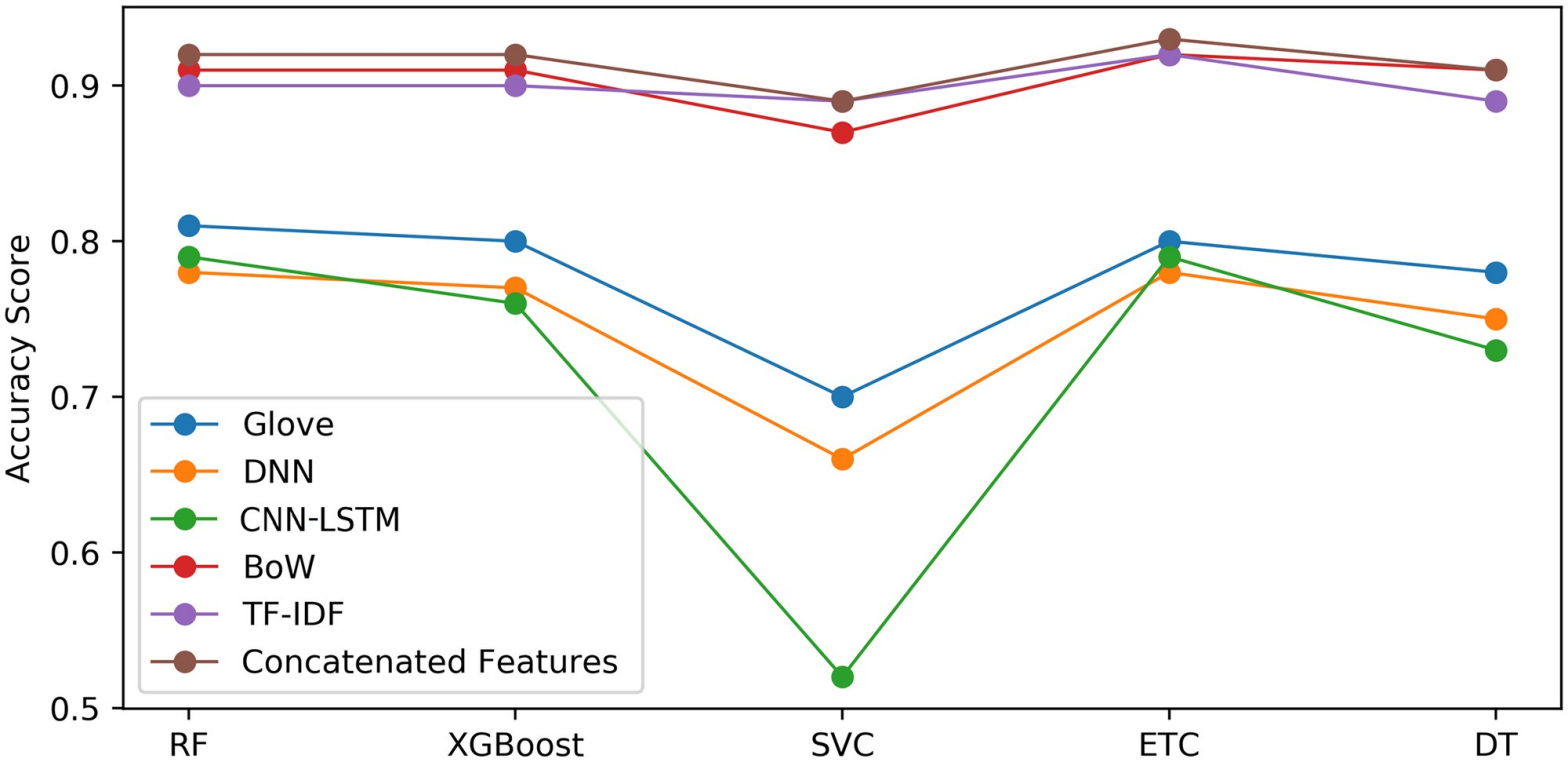
Models accuracy performance for SS2 using the concatenated feature engineering technique.

Our concatenation feature engineering approach outperforms the GloVe, the CNN-LSTM and the DNN features for all models in accuracy [57]. This is due to the fact that GloVe constructs the feature set on the basis of co-occurrence of features, which as evidence is not concrete and only probabilistic. Thus, the training of machine learning models remains incomplete and insufficient. This fact is pointed out [58]. The recommendation is to train the deep learning approaches with extensive data so that resultant co-occurrence distributions converge. The Fig 9 shows detailed results of the five used models.

### 5.5 Results using Long Short-Term Memory

We also compared the performance of LSTM with that of the machine learning models. The LSTM uses an architecture of the embedding layer between the input layer and the LSTM layer, which creates a vector of input text features for the LSTM layer. A dense layer is created with 256 neurons activation function ‘relu’ (Rectified Linear Unit). Next, a dropout layer with a 0.5 dropout rate is used to drop the neurons during training, thus reducing the chances of over-fitting the model. The output layer with the sigmoid function is used to find the probability of each class. Our LSTM model shows poor performance on the selected dataset by attaining an accuracy score of 0.577. This poor performance is due to the fact that the dataset is too small for a deep learning model. This is in accordance with the study that reports the poor performance of deep learning models on small datasets [39, 58]. We also used the BiLSTM [59] and CNN-LSTM [57] and achieved the accuracy score of 0.579 and 0.61 reflectively. BiLSTM and CNN-LSTM take more training time as compared to LSTM but does not give much improvement. There is a possibility of the unsuitability of the used architecture for LSTM and other models for the selected dataset. Hence, the results cannot be conclusive without further investigation of LSTM on other datasets or with more tuned architecture.

## 6 Conclusion

This work reports a methodical investigation of newly emerged Covid-19 sentiments as expressed in tweets by users. We used preprocessing techniques and features extraction techniques to train the machine learning model using the 80% data (6022 Tweets) and evaluate its performance using the remaining 20% data (1506 Tweets). The study concludes that ETC is the best performer with our features concatenation engineering approach.

RQ 1 posed investigation of the comparative performance of five supervised machine learning models, RF, XGBoost, SVC, ETC, and DT for Covid-19 sentiments. We addressed this question by acquiring a tweets dataset, cleaning it, and finding its sentiment scores using TextBlob. TF-IDF and BoW are used for feature extraction. Out of five trained and tested models, ETC outperforms others. This is due to the fact that linearly separable class planes are not possible for textual data such as tweets. Thus models working on discrete states tend to lose accuracy, which is indirectly covered by implicit randomization during ETC. For the sake of completeness, we also trained and tested one deep learning model, LSTM. It exhibits the lowest performance. It is understandable due to the small dataset, which does not allow enough learning paths for an overall stable system that can handle test points.

RQ 2 posed investigation of effects of an engineered feature set. This question is addressed by proposing a feature set by concatenation of TF-IDF and BoW. Again our proposed feature set outperforms the two standard techniques, TF-IDF and BoW. This is due to the fact that an extended feature set allows more training points, thus increasing the chances of the test point to lie within closer proximity of one of those training points.

We compared the performance of models using sentiment scores generated using TextBlob and Vader. Of all three feature extraction techniques used, TF-IDF, BoW, and concatenation, our approach gave better results. This is mostly due to the fact that Covid-19 tweets do not have much spread in story-lines. Of those few features extracted, there is a trend of normalized distribution, hence keeping Vader at a disadvantage. Lastly, we also compared the performance of models built on top of our winner feature technique, the concatenated set, and GloVe. Again, our proposed technique performs better. This can be associated with the complexity that emerges due to all possible co-occurrence of features. This complexity actually affects the correct decidability of most of the used supervised learning models.

In future work we plan to direct our work towards the deep learning approaches, specifically to improve their performance on small datasets.
